# Potential Role of Foliar Application of *Azotobacter* on Growth, Nutritional Value and Quality of Lettuce under Different Nitrogen Levels

**DOI:** 10.3390/plants11030406

**Published:** 2022-02-01

**Authors:** Zahra Razmjooei, Mohammad Etemadi, Saeid Eshghi, Asghar Ramezanian, Faezeh Mirazimi Abarghuei, Javad Alizargar

**Affiliations:** 1 Department of Horticultural Science, School of Agriculture, Shiraz University, Shiraz 71441-65186, Iran; zahra.razmjooii1995@gmail.com (Z.R.); eshghi@shirazu.ac.ir (S.E.); ramezanian@shirazu.ac.ir (A.R.); mirazimi_f@yahoo.com (F.M.A.); 2 Research Center for Healthcare Industry Innovation, National Taipei University of Nursing and Health Sciences, Taipei 112, Taiwan; 3 School of Nursing, National Taipei University of Nursing and Health Sciences, Taipei 112, Taiwan

**Keywords:** *Azotobacter*, biostimulants, nitrate accumulation, nitrogen supplements, proline content

## Abstract

Vegetables can be treated with biofertilizers as an alternative to chemical fertilizers because of their low toxicity. We investigated the effects of foliar spraying of *Azotobacter* under different levels of nitrogen (100, 150 and 200 mg/L in nutrient solution) on the growth, nutritional value, nitrate accumulation and antioxidant enzyme activities of hydroponically grown lettuce. The experiment was laid out in a completely randomized design with four replicates in a factorial combination. Plants treated with *Azotobacter* and 200 mg/L nitrogen had greater leaf area and photosynthetic pigments than plants treated with 200 mg/L nitrogen without spraying with *Azotobacter*. Increasing nitrogen levels increased leaf number, fresh and dry weights, leaf area and nitrate accumulation in lettuce plants. Peroxidase (POD) activity increased by 95.4% at a nitrogen level of 200 mg/L compared to a nitrogen level of 100 mg/L. Ascorbate peroxidase (APX) activity and leaf phosphorous (P) and potassium (K) concentrations were the highest in plants treated with a nitrogen source of 100 mg/L without foliar application of *Azotobacter*. As nitrogen levels increased in all treatments, nitrate reductase (NR) activity decreased and reached a minimum at the 200 mg/L nitrogen level. In general, foliar application of *Azotobacter* sp. can be used to promote plant growth and reduce nitrate accumulation in lettuce.

## 1. Introduction

In agricultural areas, modern technologies such as soilless cultured crops, hydroponics and aquaponics are used for vegetable and herb production [1]. This cropping system has many advantages including the ability to recycle water and nutrients, protect the environment and improve the quality and quantity of the crop [2].

Lettuce (*Lactuca sativa* L.) is one of the most important leafy vegetable crops worldwide. It provides a considerable amount of polyphenolic compounds, vitamins A, C and E, calcium and iron when consumed as fresh green salad [3]. The continued use of nitrogen fertilizers has increased the nitrate content in vegetable leaves without improving output, raising the risk of agricultural pollution and negatively affecting human health [4]. However, to overcome these problems, there is an alternative method of agricultural management, which is often referred to as a challenge for sustainability in agriculture, and ecologically sound means to reduce the use of inorganic fertilizers by organizing programs to increase agricultural productivity, especially in vegetable production. Therefore, clean techniques such as biostimulants are considered an alternative source of nutrition for production [1,5].

Previous research has extensively investigated the use of plant growth promoting rhizobacteria (PGPR) as biofertilizers to promote plant growth [6,7,8,9]. Foliar and root application of PGPR has been shown to increase macro- and micronutrient content, induce systemic resistance to pathogens and withstand environmental stress conditions [9,10,11].

In addition, microorganisms such as Pseudomonas, *Azotobacter* and *Azospirillum* can produce different types of plant hormones [2,11,12]. In the PGPR group, *Azotobacter*, which belongs to the Gammaproteobacteria group, can fix atmospheric nitrogen and grow effectively under nitrogen-free conditions. They synthesize cellular proteins by utilizing atmospheric nitrogen. The availability of nitrogen is related to cell death after mineralization of the cellular protein [13]. Moreover, *Azotobacter* strains showed positive effects on plant growth, crop yield and nitrogen requirement of horticultural crops and achieved significant yield increases (up to 40%) [6,11,14]. Ahmed et al. [12] found that treatment of lettuce plants with *Azotobacter chroococcum* and *Azospirillum lipoferum* as biofertilizers resulted in significant increases in aerial plant height, leaf number and fresh weight. A significant decrease in nitrate accumulation was observed when the plants were treated with biofertilizer. Foliar application of nitrogen fixing bacteria, namely *Azotobacter*, *Azospirillum* and *Beijerinckia*, resulted in improved leaf quality as specified by their protein content and effect on breeding in mulberry plants [6]. *Azotobacter* spp. and *Azospirillum* spp. showed significant increases in chlorophyll content in hydroponically grown strawberries under different nitrogen concentrations [11].

PGPR are highly effective in promoting plant growth. However, information on the effects of foliar application of PGPR on plant growth, physicochemical properties and nitrate accumulation in greenhouse lettuce in a soilless crop with different nitrogen levels is lacking and inconclusive. Therefore, this study aimed to investigate the effects of foliar spraying of *Azotobacter* on growth parameters, antioxidant enzyme activities and macronutrient content of hydroponically grown lettuce with different levels of nitrogen in nutrient solution. This is the first report on the effects of foliar application of PGPR on lettuce cultivation.

## 2. Results

### 2.1. Morphological Parameters

**Figure 1:**
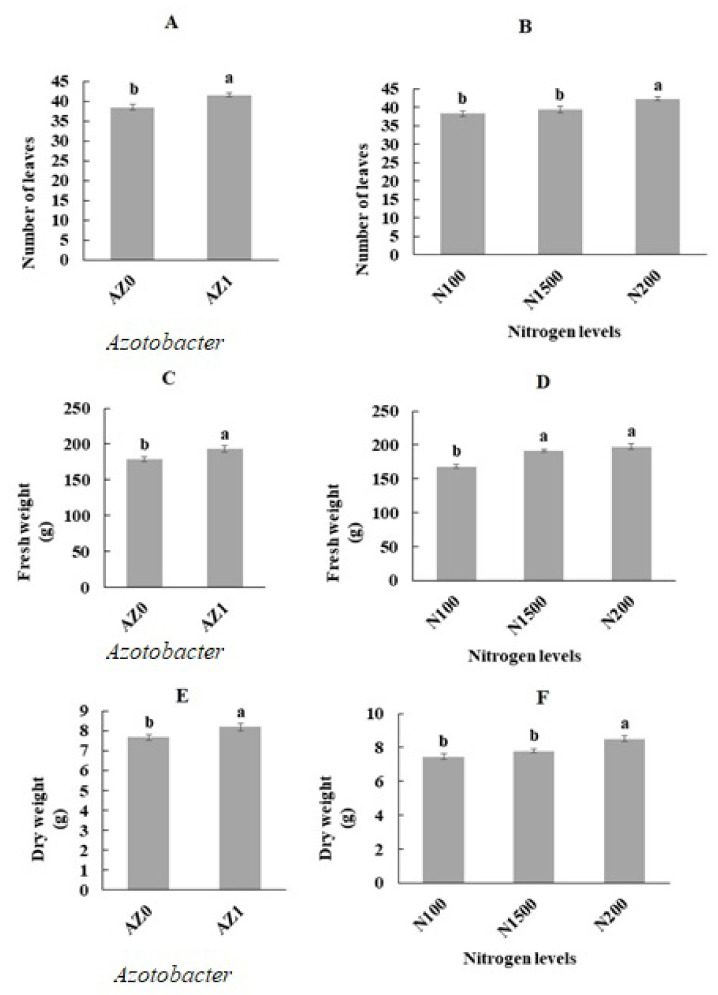
Leaf number, fresh and dry weight of aerial parts in hydroponically grown lettuce influenced by *Azotobacter* (**A**,**C**,**E**) and under different nitrogen levels (**B**,**D**,**F**). The letters indicate the mean comparison using LSD test (*p* < 0.05) which was implemented for examining the differences among the treatments.

**Figure 2:**
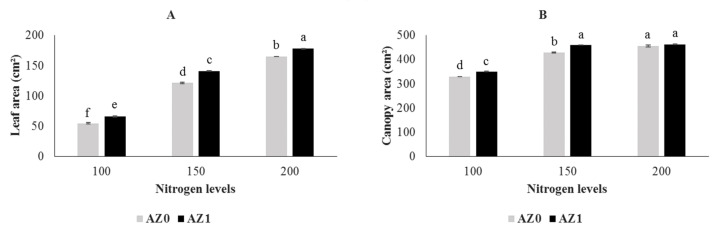
Leaf area (**A**) and canopy area (**B**) in hydroponically grown lettuce influenced by *Azotobacter* under different nitrogen levels. The letters indicate the mean comparison using LSD test (*p* < 0.05) which was implemented for examining the differences among the treatments.

The effects of bacterial application and different nitrogen ratios on leaf number, fresh weight and dry weight of aerial parts of lettuce plants were significant at *p* < 0.01 (Table 1). Leaf and canopy areas showed significant differences in lettuce plants sprayed with *Azotobacter* in combination with different nitrogen concentrations under hydroponic culture (*p* < 0.001) (Table 2). Foliar spraying with *Azotobacter* significantly improved plant growth such as the number of leaves, fresh weight and dry weight of aerial parts (Figure 1A,C,E). As shown in Figure 1B, the 100 and 150 mg/L nitrogen treatments decreased leaf number (10.45% and 7.30%, respectively) compared to the control treatment (200 mg/L nitrogen). As expected, at the end of the experiment, the highest and lowest fresh weights of the area were observed in the 200 mg/L nitrogen (197.37 g) and 100 mg/L nitrogen treatments with a reduction of 17.22% compared to the control treatment. However, no significant (*p* < 0.001) difference was observed in the lettuce plants treated with 150 mg/L nitrogen (Figure 1D). While there was no significant (*p* < 0.001) difference in dry weight of lettuce plants treated with 100 and 150 mg/L nitrogen, the control treatment had a significant effect and the plants treated with 200 mg/L nitrogen showed a greater increase (8.85 g) than the other two treatments (Figure 1F). It is interesting to note that the leaf area increased significantly with increasing nitrogen content (Figure 2A). The results also showed that foliar application of *Azotobacter* in combination with nitrogen concentrations of 100, 150 and 200 mg/L significantly increased leaf area compared to the above nitrogen concentrations without bacterial application. Leaf area improved (7.8%) in lettuce plants treated with *Azotobacter* and 200 mg/L nitrogen compared to the control treatment (200 mg/L nitrogen without the application of bacteria). The lowest leaf area was observed in plants treated with 100 mg/L nitrogen without the application of *Azotobacter* (54.96 cm^2^). As can be seen in Figure 2B, the increase in canopy area in plants sprayed with bacteria at 100 and 150 mg/L doses of nitrogen in the nutrient solution followed a faster trend than that for the above amount of nitrogen without application of bacteria. The maximum and minimum canopy areas were observed at 200 mg/L nitrogen with bacterial inoculation (462.91 cm^2^) and 100 mg/L nitrogen without bacterial spraying (329.70 cm^2^) (Figure 2B).

### 2.2. Photosynthetic Pigments

The results of ANOVA (Table 3) show that the content of chlorophyll a, chlorophyll b, total chlorophyll and carotenoids were significantly affected by the nitrogen sources, *Azotobacter* and their interactions. As shown in Table 3, the maximum contents of chlorophyll a, b and total chlorophyll and carotenoids in foliar application of *Azotobacter* in combination with 200 mg/L nitrogen source (0.714, 0.149, 0.874 and 0.262 mg/g FW) were recorded, while the minimum levels of chlorophyll a, b, total chlorophyll and carotenoids (0.543, 0.112, 0.644 and 0.202 mg/g FW) were recorded at a nitrogen level of 100 mg/L without bacterial inoculation (Table 3). As shown in Table 3, increases of 12.64%, 12.26%, 8.04% and 7.21% were observed for chlorophyll a, b, total and carotenoids, respectively, at a nitrogen level of 200 mg/L with bacterial inoculation compared to the control (200 mg/L without bacterial inoculation).

### 2.3. Total Soluble Sugar and Starch Content

**Figure 3:**
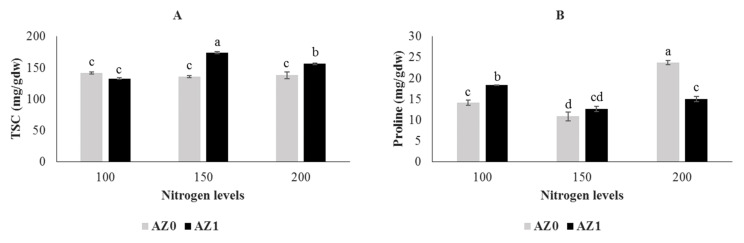
Total soluble carbohydrate (TSC) (**A**) and proline (**B**) contents in hydroponically grown lettuce influenced by *Azotobacter* under different nitrogen levels. The letters indicate the mean comparison using LSD test (*p* < 0.05) which was implemented for examining the differences among the treatments.

The data showing the effects of the biofertilizer and the various nitrogen levels, both individually and in combination, on total soluble carbohydrate are shown in Table 4. All nitrogen contents, bacteria and their combinations significantly (*p* < 0.001) improved the total soluble sugar content of lettuce compared to their respective controls (data not shown). Foliar application of *Azotobacter* in combination with 150 and 200 mg/L nitrogen significantly improved the total soluble sugar content by 26.04% and 13.18%, respectively, as compared to the control. The highest soluble total sugar content was recorded at a nitrogen level of 150 mg/L with the application of bacteria (174.01 mg/g DW). *Azotobacter* inoculation combined with 100 mg/L nitrogen had the lowest total soluble sugar (132.85 mg/g DW) (Figure 3A). The ANOVA results (Table 4) show that the starch content was not affected by the biofertilizer and the different nitrogen contents, either individually or in combination.

### 2.4. Proline and Protein

Figure 3B shows that the maximum proline content (132.85 mg/g DW) was recorded when plants were fertilized with 200 mg/L nitrogen source without inoculation with *Azotobacter* (control) (23.76 mg/g DW). An increase of 30.07% in leaf proline accumulation was observed at a nitrogen level of 100 mg/L with bacterial inoculation compared with the nitrogen level without the application of bacteria (Figure 3B). According to the ANOVA result, the protein content was not influenced by *Azotobacter* and different nitrogen levels, both individually and in combinations (Table 4).

### 2.5. Secondary Metabolites: Total Phenol Content, Total Flavonoid Content and Antioxidant Activity

**Figure 4:**
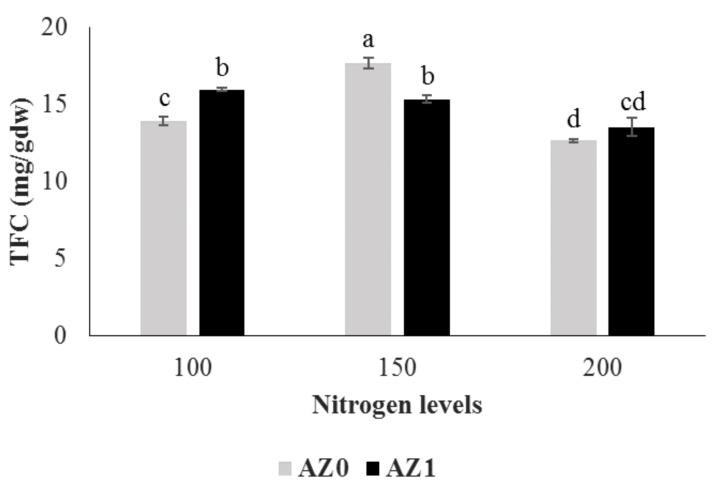
Total flavonoid content (TFC) in hydroponically grown lettuce influenced by *Azotobacter* under different nitrogen levels. The letters indicate the mean comparison using LSD test (*p* < 0.05) which was implemented for examining the differences among the treatments.

The results of ANOVA showed that total phenolic content and antioxidant activity were not affected by *Azotobacter* and different nitrogen levels, both individually and in combination (Table 5). Total flavonoid content was highest in plants treated with 150 mg/L nitrogen source without *Azotobacter* inoculation compared to the other treatments and control. Foliar application of *Azotobacter* significantly (14.45%) increased the total flavonoid content at nitrogen level of 100 mg/L compared to the said nitrogen level without bacterial application, while there was no significant difference with the control treatment (Figure 4).

### 2.6. Antioxidant Enzyme Activity 

**Figure 5:**
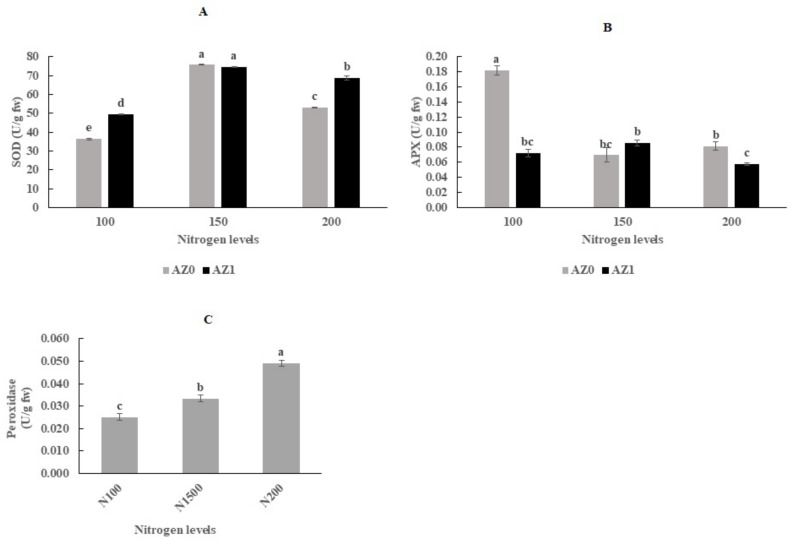
Antioxidant enzyme activity, SOD (**A**), APX (**B**) and POD (**C**) in hydroponically grown lettuce influenced by *Azotobacter* under different nitrogen levels. The letters indicate the mean comparison using LSD test (*p* < 0.05) which was implemented for examining the differences among the treatments.

The results of ANOVA showed that the activity of CAT was not affected by *Azotobacter* and different nitrogen contents, both individually and in combination, while the enzyme activity of POD was affected exclusively by different nitrogen contents (Table 6). For the activity of SOD, while no differences were observed between 150 mg/L nitrogen source without *Azotobacter* inoculation and 150 mg/L nitrogen source in combination with *Azotobacter* inoculation, both treatments showed the highest enzyme activity (Figure 5A). Plants fertilized with 100 and 200 mg/L nitrogen and sprayed with *Azotobacter* showed higher SOD enzyme activity than plants fertilized with the above amounts without bacterial inoculation (35.86% and 29.82%, respectively). The highest APX enzyme activity was recorded in plants treated with 100 mg/L nitrogen source without inoculation of *Azotobacter* (Figure 5B). The POD antioxidant enzyme activity increased sharply after the addition of nitrogen sources and reached the maximum at 200 mg/L nitrogen. As shown in Figure 5C, POD enzyme activity increased by 95.4% at 200 mg/L nitrogen compared to 100 mg/L nitrogen.

### 2.7. Nutrient Elements

The mineral concentrations in lettuce plant as a function of leaf bacterial application at different nitrogen levels were presented in Table 7. Leaf N concentration was significantly affected by *Azotobacter* application at different nitrogen concentrations. The lowest leaf N concentrations were observed in plants fertilized with 100 mg/L nitrogen without bacterial inoculation (2.2%) and the control treatment resulted in the highest (3.6%). According to the results of ANOVA (Table 7), P and K concentrations in leaves were significantly affected by different nitrogen levels, *Azotobacter* and their interactions. P and K concentrations in leaves of plants treated with nitrogen level of 100 mg/L without bacterial inoculation (0.4% and 4.008%, respectively) were higher than those in plants with other treatments and control. In general, P and K content of leaves decreased with increasing nitrogen content in nutrient solution (Table 7).

### 2.8. Nitrate Accumulation and Nitrate Reductase Activity

**Figure 6:**
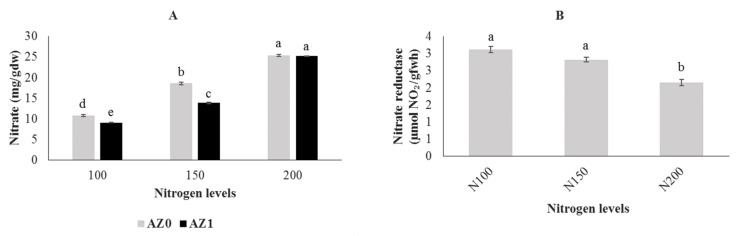
Nitrate accumulation (**A**) and nitrate reductase activity (**B**) in hydroponically grown lettuce influenced by *Azotobacter* under different nitrogen levels. The letters indicate the mean comparison using LSD test (*p* < 0.05) which was implemented for examining the differences among the treatments.

The data showing the effects of biofertilizer and different amounts of nitrogen both individually and in combination on nitrate accumulation and nitrate reductase activity are shown in Table 8. Foliar application of *Azotobacter* at different nitrogen levels reduced nitrate accumulation compared to application without bacteria, while no significant differences were observed between inoculation of *Azotobacter* at a nitrogen source of 200 mg/L and the control. The maximum and minimum nitrate levels were observed in the control treatment and in the treatment with 100 mg/L nitrogen combined with *Azotobacter* application, respectively (Figure 6A). Nitrate reductase activity decreased with increasing nitrogen content in all treatments and reached a minimum in the control treatment (Figure 6B). Plants fertilized with 100 and 150 mg/L nitrogen showed an increase in nitrate reductase activity by 44.65% and 31.16%, respectively, as compared to control (Figure 6B).

## 3. Discussion

Nitrogen (N), one of the most important macronutrients, significantly determines crop yield and quality [15]. Moreover, several species have been reported to have enhanced growth and development in plants fertilized with different nitrogen concentrations or inoculated with PGPR in hydroponics [11,16,17,18,19]. The data obtained showed that the effects of different nitrogen concentrations on lettuce sprayed with PGPR were consistent with previous reports, with plant growth showing an increasing trend [11,20]. *Azotobacter* sprayed lettuce increased vegetative growth expressed in number of leaves, fresh and dry weight of head, leaf and canopy area. Similar results were reported by Al-Taey and Majid (2018) [21] for lettuce and by Al-Taey et al. [1] for broccoli. Sudhakar et al. [6] found that foliar application of biofertilizers, especially *Azotobacter*, significantly improved the growth parameters of mulberry plants compared to the untreated plants. The positive effect of *Azotobacter* on plant growth can be attributed to the fixation of sufficient nitrogen on the leaf surface, production of phytohormones such as auxins, gibberellins and cytokinins that alter plant growth and morphology, and its antagonistic effect on fungi and plant pathogenic bacteria [6,11,14,17,18,22]. Accordingly, this is the first study on the use of nitrogen-fixing bacteria by foliar spraying in lettuce under soilless culture. Foliar application of *Azotobacter* improved the availability of leaf nutrient content and some growth-promoting substances, which contributed to an increase in leaf number, leaf area, fresh weight and dry weight of head, and canopy area of soilless grown lettuce. These desirable conditions resulted in higher photosynthetic activity by utilizing more light energy and CO_2_, which in turn increased the synthesis of metabolic materials and translocation, accumulation of dry matter and its content in the lettuce leaves. Similarly, growth and productivity of most vegetables are related to adequate nitrogen supply in nutrient solutions [23], which is in agreement with the current study. Tei et al., (2000) suggested that nitrogen addition in nutrient solution leads to improvement in vegetative growth and is responsible for improved leaf area and increased chlorophyll concentration as a result of impressive utilization of photosynthetically active radiation [24]. While in the present study, 200 mg/L nitrogen resulted in positive effect on vegetative growth of lettuce plant, Becker et al., (2015) found that plant growth was affected by low nitrogen concentrations [25]. Leaf and canopy area increased even further when the combination of *Azotobacter* with nitrogen fertilizer was used, which was in agreement with the study of Umar et al. [26]. The results of this study indicated that nitrogen addition in the solution and growth stimulating bacteria increased photosynthetic pigments in soilless grown lettuce. Moreover, many authors have confirmed these results [27,28,29,30]. Since chlorophyll is an important index for evaluating the growth and development of lettuce plants, the application of nitrogen (a key factor for chlorophyll formation) and *Azotobacter* appeared to promote the development of chlorophyll, its synthesis, the induction of chlorophyll-related enzymes and the activity of photosynthetic enzymes, resulting in the regulation of photosynthesis [27,29,30]. 

The increase in carotenoid content with the application of *Azotobacter* and nitrogen sources has elucidated the key role of these compounds in the photosynthetic process of lettuce growth, photosystem support, induction of carotenoid protection and prevention of carotenoid oxidation by reactive oxygen species [28,29,31].

Foliar application of growth-stimulating bacteria and different levels of nitrogen in hydroponically grown lettuce did not increase protein content, which was not consistent with the results of Pan et al., [25] and Latef et al. [28,32]. 

Increased nitrogen applications in the nutrient solution of soilless grown lettuce can increases proline accumulation in lettuce leaves [30]. The reports of Sánchez et al. also confirmed that the accumulation of this nitrogenous form can act as osmolyte and other important cellular functions [33]. Accordingly, foliar spraying of *Azotobacter* reduced proline content in lettuce leaves when treated with 200 mg/L nitrogen. It appeared that the application of bacteria led to a reduction of this nitrogen excess by improving the availability of leaf nutrient content and some growth promoters. The higher nitrogen content in combination with *Azotobacter* resulted in a significant increase in soluble sugar in the lettuce leaves. This increase in total soluble sugar is due to the enhancement of the photosynthetic system of the host plants, which is in agreement with the study of Arough et al. [13] in triticale.

Secondary metabolites such as phenols and flavonoids are considered antioxidants that protect against oxidative damage and play an important role in human nutrition [16,20]. In agreement with previous findings [34], the present results again showed that antioxidant activity and total phenolic content, the most important quality variables in lettuce plants, were not affected by nitrogen content. Nevertheless, the changes in total phenolic content and antioxidant activity are associated with increased nitrogen applications as reported by Mampholo et al. [16]. Flavonoid content showed a semi-downward trend with increasing nitrogen application rates in lettuce plants. This confirms earlier findings of Qadir et al. [32], who concluded that increasing nitrogen application rates led to a decrease in flavonoid content of lettuce [35]. 

While reactive oxygen species are vital in host defense, ROS residues can induce oxidative damage [36]. SOD, POD, APX and CAT are antioxidant enzymes that are critical in detecting and detoxifying excess reactive oxygen species, and their activities may be elevated following exposure to moderate environmental stresses [36,37,38]. In our investigation, the activities of SOD and POD rose significantly as nitrogen (N) levels increased. SOD activity, on the other hand, increased with increasing N concentration up to a specific level (150 mg/L). According to Liao et al. [39], the activity of these enzymes may rise at low levels of N supplementation but may be suppressed at high levels. Changes in SOD activity caused by *Azotobacter* treatment may potentially be due to altered synthesis and accumulation of less active enzymes, as well as higher SOD turnover [40,41]. The increase in antioxidant enzyme activity did not relieve ROS toxicity, resulting in photosynthesis damage and chlorophyll degradation. According to Wang et al. [42], appropriate N dosage can improve the activity of antioxidant enzymes, increase the scavenging ability of mesophyll cells against ROS and maintain cell integrity, all of which increase yield [42]. 

Several studies indicate that the physical and chemical properties of the growth substrate and inoculation with biofertilizers can influence the mineral composition of plants by increasing the content of nutrients in terms of health benefits for humans [20]. The current study is in line with previous studies which showed that nitrogen content in leaves increases in line with nitrogen supply in the nutrient solution [43].

It is interesting to note that P and K content peaked at the lowest nitrogen additions in hydroponically grown lettuce. It seems that the increase in leaf area along with the dilution of minerals as the nitrogen content of the solution increases contributes to ion antagonism and decrease in mineral concentration [15]. 

Nitrate accumulation in vegetables is influenced by several factors, including nitrogen amounts and sources, light intensity and temperature and internal factors such as genotype and species [29,44]. In addition, the activity of the enzyme nitrate reductase is influenced by the nitrate availability of leaves under soilless culture. The use of lower nitrogen content in the nutrient solution resulted in a decrease in nitrate content in lettuce leaves, as previously shown by Balanz et al. [45]. The application of *Azotobacter* resulted in a decrease in nitrate content in the leaves compared to the control, possibly as a result of the utilization of nitrates by the bacteria in an anaerobic respiration process [45]. The results showed that the higher nitrate concentrations in the solution did not result in higher enzyme activity. This observation differs from the reports of Wenceslau et al. [19]. It seems that not only nitrate availability affects nitrate reductase activity, but also other factors such as environmental conditions should be considered. Lower light intensity may lead to greater accumulation of nitrate in leafy vegetables by decreasing nitrate reductase activity, possibly a similar mechanism followed in the current study [44].

## 4. Material and Methods

### 4.1. Bacteria Preparation and Treatment 

Bacteria belonging to the genus *Azotobacter* sp. (endemic to Iran, from Iranian Research Organization for Science and Technology (IROST), Persian Type Culture Collection (PTCC)) were grown in nutrient broth medium (NB) at 28 ± 2 °C for 24 h on a temperature-controlled shaker (150 rpm). Bacterial density was measured at 600 nm. For inoculation with *Azotobacter* sp. an inoculum of 106 CFU/mL was prepared by centrifuging the freshly grown bacterial culture at 10,000× *g* for 5 min. The resulting suspensions were used to treat lettuce plants.

F1 seeds of lettuce cultivar ‘Armoni’ (*Lactuca sativa* L.) were sown in plastic trays and irrigated under a misting system. The seedlings were then planted at four-leaf stage in 1000 mL pots containing coco peat perlite media (50:50 *v/v*). The nutrient solution used in the experiment initially consisted of half the concentration of Hoagland and Arnon’s formulation for three weeks until the plants had 6–8 leaves, and then treatment was started [46]. The experiment was designed as a factorial combination based on a completely randomized experimental design with two factors: *Azotobacter* (foliar sprayed plants and non-sprayed plants) and 15:85, 22.5:127.5, 30:170 mg/L ammonium: Nitrate ratio (100, 150, 200 mg/L) with 4 replicates consisting of 8 plants per treatment. ‘Armoni’ lettuce was provided by Serene Company, Turkey. The nutrient solutions were sub-fertigated to the lettuce plants every other day and the pH was 5.7. The plants were grown in a greenhouse at 25 ± 3 °C during the day and 18 ± 3 °C at night. The relative humidity was 60%. Plant leaves were sprayed with diluted bacterial suspension (2000 CFU/mL) at 7-day intervals until they became wet, and nitrogen treatments were applied every other day for one month.

### 4.2. Plant Growth Measurements

Growth parameters such as number of leaves per plant were counted 7 weeks after transplanting and the average number of leaves per plant was expressed in numbers. Leaf and canopy area were determined using Image J. software [47]. Plant parts were harvested and weighed to determine fresh aerial mass. They were then oven dried at 70 °C for 48 h and dry weight (DW) was measured using a digital balance (AND GF −3000) with an accuracy of 0.01 g.

### 4.3. Chlorophyll and Carotenoid Measurements

The content of photosynthetic pigments (total chlorophyll, chlorophyll *a*, chlorophyll *b* and carotenoids) in lettuce leaves was measured according to the method described by Hiscox and Israelstam [48]. The absorbance of the extract solution was measured at 663, 645 and 470 nm using spectrophotometer (Epoch Microplate, BioTek Instruments Spectrophotometer, Winooski, VT, USA). The final content of each pigment was calculated using the following formulae and the results were expressed as mg per g fresh weight of leaves.

(1) $$\mathrm{Chlorophyll}\;a\;(\mathrm{mg}/\mathrm{gFW})=\frac{12.7\left(A_{663}\right)-2.69\left(A_{645}\right)\times V}{FW}$$

(2) $$\mathrm{Chlorophyll}\;b\;(\mathrm{mg}/\mathrm{gFW})=\frac{22.9\left(A_{645}\right)-4.68\;\left(A_{663}\right)\times V}{FW}$$

(3) $$\mathrm{Total}\;\mathrm{Chlorophyll}\;(\mathrm{mg}/\mathrm{gFW})=\frac{20.2\;\left(A_{645}\right)+8.02\;\left(A_{663}\right)\times V\;}{FW}$$

(4) $$\mathrm{Carotenoid}\;(\mathrm{mg}/\mathrm{gFW})=\frac{1000\;\left(A_{470}\right)-1.82\;C_{a\;}-85.02\;C_{b}}{198}$$

### 4.4. Total Soluble Sugar and Starch Concentrations

To determine the concentration of soluble sugar, 0.1 g of the dry leaf samples were extracted twice with ethanol (80%). The samples were centrifuged at 5000 rpm for 10 min and the supernatant was adjusted to 10 mL. Soluble sugar content was measured according to the method described by McCready et al. [49]. The starch concentration of the samples was measured using Anthrone reagent (Merck KGaA, Darmstadt, Germany) [49].

### 4.5. Proline Content 

Proline accumulation was extracted from 100 mg samples of lettuce leaves with 10 mL methanol and estimated using the ninhydrin reagent (Sigma-Aldrich, Darmstadt, Germany) according to the protocol described by Carillo and Gibon [50]. The samples were measured at 515 nm by a microplate spectrophotometer (Epoch, Biotech, Winooski, VT, USA). Methanol and the reaction mixture were used as blanks. The proline content was calculated using a standard curve with L-proline and reported as mg·g^−1^ dry weight.

### 4.6. Total Protein Determination

Protein content was measured according to the procedure described by Bradford (1976) using Bradford reagent and Coomassie Brilliant Blue (Fluka, Buchs, Switzerland). To 0.03 mL of the enzyme extract, 1.5 mL of Bradford reagent was added. Within 60 min, absorbance was measured using spectrophotometer at 595 nm against a blank (0.03 mL of suitable buffer and 1.5 mL of Bradford reagent).

### 4.7. Secondary Metabolites Determination

#### 4.7.1. Total Phenolic Content

Total phenolic content was measured in dry leaf samples using Folin-Ciocalteau reagent (Sigma-Aldrich, Darmstadt, Germany); 0.02 mL extracts of leaf samples were added to 0.02 mL Folin-Ciocalteau reagent and 0.04 mL sodium carbonate solution. Then the test tubes were shaken and stored at laboratory temperature for 30 min. After incubation, the absorbance of the mixture was measured at 790 nm. Different concentrations of gallic acid (100–1000 mg/L) were used to construct the standard curve and the results were expressed as mg gallic acid per 100 g dry weight [51].

#### 4.7.2. Total Flavonoid Content

The method described by Hussein et al. [52] was performed for the analysis of total flavonoid content with some modifications. To 100 μL of extract, 50 μL of 0.5 M NaNO_2_ solution was added followed by 50 μL of 0.5 M AlCl_3_ solution and 1170 μL of 30% methanol [52]. After adding 333 μL 1 M NaOH, the mixture was allowed to stand at room temperature for 5 min. The solution was shaken and UV absorbance at 506 nm was recorded against quercetin as reference. The result was expressed as mg/100 g DW.

#### 4.7.3. Antioxidant Activity

The 2,2-Diphenyl-1-Picrylhydrazyl (DPPH) radical scavenging assay was performed using the DPPH radical assay according to the method of Martins et al., (2016) with minor modifications. A total of 20 μL of the leaf extract was added to 800 μL of a 0.1 mM methanolic DPPH solution. The mixture was kept at room temperature for 30 min before any change in absorbance at 517 nm was observed. The percentage reduction of DPPH was defined as follows: DPPH (%) = 100 (A_0_ − A_c_)/A_0_, where A_0_ is the control absorbance without sample concentration and A_c_ is the value when the sample concentration is added.

#### 4.7.4. Antioxidant Enzyme Activity

Frozen leaf samples were used for the determination of antioxidant enzyme activity including catalase (CAT), ascorbate peroxidase (APX), superoxide dismutase (SOD) and peroxidase (POD). Leaf samples prepared according to the method of Ozden et al. [53] were used for enzyme extraction. The total activity CAT was determined as the reduction of H_2_O_2_ (extinction coefficient 39.4 mM^−1^ cm^−1^) measured at 240 nm for 1 min [53]. The 3 mL reaction mixture contained 2800 μL 50 mM phosphate buffer (pH 7.0), 100 μL 0.1 M H_2_O_2_ and 100 μL enzyme extract. Immediately after the addition of the enzyme extract to the reaction mixture, the initial linear velocity of absorbance decrease at 240 nm was recorded and the activity of CAT was calculated. The activity of CAT was calculated based on the method of Sun et al. [54] and expressed as μMol H_2_O_2_ min^−1^ mg^−1^ protein. APX activity was determined as the decrease in A290 (extinction coefficient 2.8 mM^−1^ cm^−1^) for 1 min in a 2 mL reaction mixture containing 1300 μL 50 mM potassium phosphate buffer (pH 7.0), 400 μL 0.1 M H_2_O_2_ and 300 μL enzyme extract [54]. The reaction was started by the addition of H_2_O_2_. The activity of APX was calculated according to the method of Sun et al. [55]. For SOD assay, the inhibition of photochemical reduction of nitroblue tetrazolium to formazan was measured by SOD at 560 nm. The activity of the enzyme was calculated according to the method of Giannopolitis and Ries (1977). The POD activity was measured as described by Sun et al. [54] and the absorbance of the reaction mixture (1700 μL 50 mM phosphate buffer (pH 7.0), 150 μL 20 mM guaiacol, 100 μL enzyme extract and 50 μL 40 mM H_2_O_2_) was measured at 470 nm. The reaction was started by the addition of H_2_O_2_ [54].

### 4.8. Mineral Analysis

The oven-dried (70 °C for 48 h) leaf samples were ground. The Kjeldahl method was used to determine total nitrogen (N) [56]. For the determination of macrophosphorus and potassium (P and K), the oven-dried samples were ashed at 500 °C. The ashed samples were dissolved in 5 mL of 2N hydrochloric acid and the final volume was made up to 250 mL with distilled water. Phosphorus concentration was measured by spectrophotometry (Epoch Microplate, BioTek Instruments Spectrophotometer, Winooski, VT, USA) according to the method of Barton (1948). K was determined by flame emission spectroscopy.

### 4.9. Determination of Nitrate Content

Determination of nitrate content in lettuce was done according to the method described by Zhao and Wang [57] with some modifications; 0.1 gr of ground sample was transferred to a 25 mL beaker. Distilled water was then added to obtain a volume of 10 mL [57]. The samples were kept in a closed room for one hour and centrifuged at 5000 rpm for 15 min. Then 0.2 mL of supernatant was added to 0.8 mL of salicylic acid-sulfuric acid 5% (*v/w*) and mixed well, and the mixture was allowed to stand at room temperature for 20 min. After incubation, 19 mL of 8% (*w/v*) NaOH solution (2 N) was added to each tube and the tubes were allowed to cool at room temperature (about 20–30 min) and the absorbance was recorded at 410 nm with the control as reference. The result was expressed as mg/g DW.

### 4.10. Determination of Nitrate Reductase

Nitrate reductase activity according to Sing et al. [58] with some modifications was measured [58]. Briefly, 0.3 g of fresh leaf was added to each test tube; 10 mL of the test solution consisting of 5% 1-propanol, 100 mM KH_2_PO_4_ (pH 7.5) and 30 mM KNO_3_ were added to each test tube. The samples were incubated in a water bath at 30 °C for 30 min (nitrate reductase activity was highest at this step). After incubation at 100 °C for 5 min and cooling at room temperature (to stop the activity of nitrate reductase enzyme), followed by the addition of 10 mL of colour development reagent containing sulphanilamide (1% *w*/*v* in 3N HCl) and N-(1-naphthyl) ethylenediamine dihydrochloride (0.02% *w*/*v*). The samples were incubated at room temperature for 15 min in the dark. The absorbance at 540 nm was measured using a spectrophotometer (Epoch Microplate, BioTek Instruments Spectrophotometer, Winooski, VT, USA). The activity of nitrate reductase was expressed as μMol NO_2_ h^−1^ g^−1^ FW.

### 4.11. Statistical Analysis

Data were statistically analyzed using R software (two-way ANOVA), and significant differences between treatment means were determined using the least significant difference (LSD) method at a 5% significance level. All error bars in the graphs indicate the standard error (mean ± SE).

## 5. Conclusions

The tested microorganism (*Azotobacter*) in combination with different nitrogen levels showed positive effects on vegetative growth (leaf area and canopy area), antioxidant enzyme activity, secondary metabolites and mineral nutrition of hydroponically grown lettuce. The lettuce plant with the lowest nitrogen content had the highest leaf macronutrients (P% and K%) under soilless culture. *Azotobacter* as a biofertilizer increased the growth and yield of lettuce compared to the untreated condition. These positive effects can be attributed to stimulation of the N assimilation pathway and nitrogen use efficiency. In addition, *Azotobacter* could benefit plants through biosynthesis of biologically active substances, stimulation of microorganisms, production of phytopathogenic inhibitors and improved nutrient availability of P, carbon and sulfur, which needs to be confirmed by further work. Foliar spraying of *Azotobacter* could be a financially effective and sustainable substitute to promote plant growth and nutrition and reduce nitrate accumulation in leafy vegetable crops.

## Figures and Tables

**Table 1:** Leaf number, fresh and dry weight of aerial parts in hydroponically grown lettuce influenced by *Azotobacter* under different nitrogen levels. Significant differences: *** *p* < 0.001, ** *p* < 0.01 and ns: non-significant.

	ANOVA		
Source	Leaf Number	Fresh Weight	Dry Weight
Bacteria	***	***	**
Nitrogen	***	***	***
Bacteria × Nitrogen	ns	ns	ns

**Table 2:** Leaf area and canopy area in hydroponically grown lettuce influenced by *Azotobacter* under different nitrogen levels. Significant differences: *** *p* < 0.001.

ANOVA		
Source	Leaf Area	Canopy Area
Bacteria	***	***
Nitrogen	***	***
Bacteria × Nitrogen	***	***

**Table 3:** Photosynthesis pigments content (mg/gFW) in hydroponically grown lettuce influenced by *Azotobacter* under different nitrogen levels.

Bacteria	Nitrogen Levels	Chlorophyll *a*	Chlorophyll *b*	Total Chlorophyll	Carotenoid
No-*Azotobacter*	100	0.543 c	0.112 d	0.644 e	0.202 d
	150	0.554 c	0.121 c	0.706 cd	0.207 d
	200	0.633 b	0.133 b	0.808 b	0.243 b
*Azotobacter*	100	0.611 b	0.133 b	0.750 bc	0.230 c
	150	0.553 c	0.126 bc	0.682 de	0.210 d
	200	0.714 a	0.149 a	0.874 a	0.262 a
signification					
Bacteria		***	***	**	***
Nitrogen		***	***	***	***
Bacteria × Nitrogen		*	**	*	*

The letters next to the numbers indicate the mean comparison using LSD test (*p* < 0.05) which illustrates for the significant interactions among treatments. The number of stars indicates the rate of significant differences among the data within the same column. Significant differences, *** *p* < 0.001, ** *p* < 0.01, * *p* < 0.05.

**Table 4:** Total soluble carbohydrate, starch, proline and protein contents in hydroponically grown lettuce influenced by *Azotobacter* under different nitrogen levels. The mean comparison using LSD test (*p* < 0.05) was implemented for examining the differences among the treatments. Significant differences: *** *p* < 0.001 and ns: Non-significant.

ANOVA				
Source	Total Soluble Carbohydrate	Starch	Proline	Protein
Bacteria	***	ns	ns	ns
Nitrogen	***	ns	***	ns
Bacteria × Nitrogen	***	ns	***	ns

**Table 5:** Total phenol content, total flavonoid content and antioxidant activity in hydroponically grown lettuce influenced by *Azotobacter* under different nitrogen levels. Significant differences: *** *p* < 0.001 and ns: Non-significant.

ANOVA			
Source	Total Phenol Content	Total Flavonoid Content	Antioxidant Activity
Bacteria	ns	ns	ns
Nitrogen	ns	***	ns
Bacteria × Nitrogen	ns	***	ns

**Table 6:** Antioxidant enzyme activity (CAT, SOD, APX and POD) in hydroponically grown lettuce influenced by *Azotobacter* under different nitrogen levels. The mean comparison using LSD test (*p* < 0.05) was implemented for examining the differences among the treatments. Significant differences: *** *p* < 0.001 and ns: Non-significant.

ANOVA				
Source	CAT	SOD	APX	POD
Bacteria	ns	***	***	ns
Nitrogen	ns	***	***	***
Bacteria × Nitrogen	ns	***	***	ns

**Table 7:** Macro nutrient contents (%) in hydroponically grown lettuce influenced by *Azotobacter* under different nitrogen levels.

Source	Nitrogen Levels	Nitrogen	Phosphorous	Potassium
No-*Azotobacter*	100	2.26 d	0.40 a	4.01 a
	150	2.64 c	0.31 c	3.34 b
	200	3.67 a	0.22 d	2.26 d
*Azotobacter*	100	2.66 c	0.34 b	3.45 b
	150	2.91 b	0.28 c	2.79 c
	200	2.92 b	0.23 d	2.42 d
signification				
Bacteria		ns	*	***
Nitrogen		***	***	***
Bacteria × Nitrogen		***	*	***

The letters next to the numbers indicate the mean comparison using LSD test (*p* < 0.05) which illustrates for the significant interactions among treatments. The number of stars indicates the rate of significant differences among the data within the same column. Significant differences, *** *p* < 0.001, * *p* < 0.05 and ns: Non-significant.

**Table 8:** Nitrate accumulation and nitrate reductase activity in hydroponically grown lettuce influenced by *Azotobacter* under different nitrogen levels. Significant differences: *** *p* < 0.001 and ns: Non-significant.

ANOVA		
Source	Nitrate Content	Nitrate Reductase Activity
Bacteria	***	ns
Nitrogen	***	***
Bacteria × Nitrogen	***	ns

## Data Availability

The data presented in this study are available on request from the corresponding author. The data are not publicly available due to the privacy statement in the original project.
